# Monthly Summary

**Published:** 1879-09

**Authors:** 


### MONTHLY SUMMARY.

*Remedies in Headache.*—Dr. W. H. Day makes the following remarks on remedies for headache :—

Croton-chloral has been recommended by Dr. Liebreich, of Berlin, as possessing a special action on the sensory branches of the fifth nerve. It is of most benefit in facial neuralgia, relieving pain and producing sleep. I have known it to prove very serviceable in some cases of nervous headache in which the disorder has chiefly occupied one temple, the occiput and neck, or one parietal bone; and in other cases not only to utterly fail, but to indure sickness and nausea, if they did not previously exist. I generally give ten grains for a dose, in plain water, though it has been recommended to dissolve the remedy in a few drops of glycerine and then add the required quantity of cinnamon water, which, to some extent, disguises the bitter, nauseous taste. On this account, it may be given in the form of a pill, beginning with two grains and increasing the dose according to the urgency of the symptoms.

Hydrate of chloral is a remedy which has become one of the most frequently chosen of therapeutic agents, from its hypnotic effects. In large doses it is narcotic, and in very large doses it is said to produce anæsthesia. In nervous and spasmodic diseases, as puerperal convulsions, trismus nascentium, and the sleeplessness of chorea, we all recognize its value. In headaches, whether of the nervous or vascular type, when the pain is wearing out the patient and sleep cannot be obtained, chloral is of undoubted value. Chloral is a remedy requiring extreme caution in its administration whenever headache has recurred so often as to exhaust the patient's nerve force. Where the pulse has become small and weak, through constitutional debility or failure of the heart's action, a full dose may be followed by syncope. Incautiously administered, it may reduce the frequency of the respiration and annihilate the pulse. It lessens blood-pressure by its action on the vaso-motor system, and also by depressing the heart's action; hence it requires caution in cases where the patient, although severely suffering, is exhausted. In some cases, after its administration, I have observed such profound sleep and quiet and shallow respiration as to make me cautious in employing it where the circulation is feeble.

Chloral has a decided effect upon the vascular system, and if the pulse be firm or hard, the face flushed, or the excitement considerable, it relaxes the muscular walls of the arteries and reduces blood-pressure. It has been recommended not to employ a larger initial dose than ten grains; but I have never witnessed any effects of an overdose from twice the quantity. Very alarming symptoms have followed doses of forty and even thirty grains, so that its use requires caution, if the nerve power be much reduced.

Gelseminum is something useful in neuralgic headache and the neuralgia arising from decayed teeth. The powder and the tincture are the two forms for administration. The dose of the former is from one to two grains, and of the latter from ten to twenty minims. It is a powerful remedy, and, as many fatal cases are recorded from an overdose, its use requires caution. My experience of it is very limited, from having many more remedies at my command with which I am better acquainted,

In one case I gave a grain of the powder in a pill every night, for sleeplessness caused by neuralgic headache, and it exercised a most beneficial effect.

Phosphorus is one of the most important agents we possess in nervous exhaustion, and its efficacy is undoubted when administered in an unoxidized state, capable of being readily assimilated. No remedy requires more care in prescribing than this; for, while in small doses it is a gentle stimulant and tonic, in large doses it depresses the heart's action, like chloral, and is not free from danger.—*British Med. Journal.*

---

*Teeth Set on Edge.*—Dr. G. Dubelle intelligently writes to the *Druggists Circular* that all acid foods, drinks, medicines, tooth washes, and powders are very injurious to the teeth. If a tooth is put in cider, vinegar, lemon juice or tartaric acid, in a few hours the enamel will be completely destroyed, so that it can be removed by the finger nail as if it were chalk. Most have experienced what is commonly called teeth set on edge. The explanation of it is, that the acid of the fruit that has been eaten has so softened the enamel of the tooth that the least pressure is felt by the exceedingly small nerves which pervade the thin membrane which connects the enamel and the bony part of the tooth. Such an effect cannot be produced without injuring the enamel. True, it will become hard again when the acid has been removed by the fluids of the mouth, just as an egg shell that has been softened in this way becomes hard again by being put in water. When the effect of sour fruit on the teeth subsides they feel as well as ever, but they are not as well; and the oftener it is repeated, the sooner the disastrous consequences are manifested.

---

*Sure Antidote to Poison.*—Dr. G. Dubelle says, if a person swallows any poison whatever, or has fallen into convulsions from having overloaded the stomach, an instantaneous remedy, most efficient and applicable in a large number of cases, is a heaping teaspoonful of common salt, and as much ground mustard, stirred rapidly in a teacupful of water, warm or cold, and swallowed instantly. It is scarcely down before it begins to come up, bringing with it the remaining centents of the stomach; and lest there be any remnant of the poison, however small, let the white of an egg or a teaspoonful of strong coffee be swallowed as soon as the stomach is quiet, because these very common articles nullify a larger amount of virulent poisons than any medicines.—*Druggists Circular.*

*Diseases of the Nervous System—Their Physiognomy.*—Dr. W. T. Gairdner says that in all diseases of the nervous system it is of paramount importance to observe the attitude and bearing of the patient, his manner of answering questions, of putting out his tongue, speaking, eating, handling familiar objects, walking, etc. In all disorders attended with paralysis, tremor or convulsion, there will be at some time or other visible phenomena affecting one or other of the modes of ordinary action just enumerated; or there may be deficient power of evacuating or of retaining the excretions of the bowels and bladder. A slight tremor of the lips, and a hesitating utterance, as if the lips and tongue had no grip over the consonants, will, along with a peculiarity in the gait, an unusual stillness in the muscles of expression, and a slight disparity of the pupils, reveal with almost certainty an early stage of one of the most hopeless of diseases—general paralysis of the insane.

A similar but more complete absence of facial expression, without any of the other characteristics just mentioned, unless it be a flaw in the articulation absolutely limited to the labial consonants, will give the key to a more rare but far less dangerous disorder—double or bilateral paralysis of the portiodura; while a one-sided action of the face and brow, with a permanently open or half open eye on the side of the paralysis, and a twist of the mouth toward the opposite side, will show forth the much more common and equally isolated paralysis of the portiodura on one side only. An open mouth, dribbling saliva, an awkwardly moving or nearly motionless tongue, with very indistinct articulation, will reveal the labioglosso-laryngeal paralysis of Trousseau and Duchenne.—*Clin. Diagnosis.*

---

*Method of Removing Hairs.*—A writer in the Michigan *Medical News* gives the following method of eradicating superfluous hairs, as practiced by Dr. C. E. Mitchell, of St. Louis. The instruments required are, a pair of good ordinary forceps to seize the hair with, then a sharp needle, fastened to a small handle (a small, sharp pegging awl will do,) which is to be passed down parallel to the hair, within its sheath, till you reach the bottom of the follicle, then withdraw: melt some nitrate of silver in an ordinary spoon, and into this fluid hold the point of a small needle (similarly fixed as the puncturing needle,) till the silver crystallizes around the point like a minute ball, then insinuate this miniature caustic point into the previous puncture, cauterize the follicle of the hair bulb or root, remove all instruments, and in a few hours the hair will ulcerate out by the roots, causing no pain or lesion, with an entire removal of hairs, avoiding all possibilities of scrubby, dwarfish returning hairs.—*Med. and Surg. Reporter.*

*Neuralgia.*—Dr. Francis Pirotte gives numerous observations in which neuralgia was cured by the spray of chemically pure ether; the pains the most unyielding to all other means, ceased almost immediately. The ether is sprayed upon the painful part two or three minutes at a time, repeating it, if necessary, two, three, or four times, allowing sufficient time between each operation for the ether to completely evaporate. The temperature of the part at once falls several degrees below zero, the skin becomes blanched, insensible, and soon the subjacent parts become equally in a state of anæsthesia, and relief follows. Sometimes, the pain flies from one spot to another, but must be followed up. No unpleasant sensation follows its use, unless the ether be impure, when annoying sensation of burning and irritation is produced. It may be employed for pains from whatever cause, itching, cholera cramps, local inflammations, tetanus, rheumatism, burns, etc. Its advantages are facility of execution, immediate disappearance of the pains, and prompt cure, and economy.

In symptomatic neuralgia, it is advisable to aid the security and the rapidity of the treatment, by appropriate internal and external measures, as sulphate and valerianate of quinia, iron, tonic bitters, iodide of potassium, removing tumors, healing wounds or ulcers, etc.—*Dental News.*

---

*Virchow on the Germ Theory.*—In his late work on "Infectious Diseases in the Army," Prof. Virchow does not anticipate much to diagnosis or treatment from microscopic investigations of *vibrios, monads, micrococci, bacteria,* etc., with reference to peculiar forms of disease. He emphasizes the fact, however, that Billroth's supposed mother plant of all these—*Cocobacteria septica*—is always present in the human body itself, notably in the intestinal canal of healthy persons; so that a primary importation, an infection, or a transference, seems hardly required; it is already at hand, and wants only favorable conditions for propagating and further growing.—*Med. and Surg. Reporter.*

---

*Chloral as a Counter Irritant.*—Made into a mass with gum tragacanth, spread on paper and applied to the skin, it will produce a blister without pain. Applied as a powder, on cotton, it causes a painful burning sensation. By the former method, a portion is absorbed and the patient falls asleep. Its action is not so uniform as cantharides, but it is a mild vesicant, or an agreeable revulsive. The author quoted would commend such "chloral paper" to physicians, the more so, as it will keep for months without losing its activity if well prepared.—*Druggists Circular.*

